# Advancing cell surface modification in mammalian cells with synthetic molecules

**DOI:** 10.1039/d3sc04597h

**Published:** 2023-11-10

**Authors:** He Yang, Lihua Yao, Yichen Wang, Gaojian Chen, Hong Chen

**Affiliations:** a College of Chemistry, Chemical Engineering and Materials Science, Soochow University 199 Ren'ai Road Suzhou 215123 Jiangsu P. R. China gchen@suda.edu.cn chenh@suda.edu.cn; b Center for Soft Condensed Matter Physics and Interdisciplinary Research, Soochow University Suzhou 215006 Jiangsu P. R. China

## Abstract

Biological cells, being the fundamental entities of life, are widely acknowledged as intricate living machines. The manipulation of cell surfaces has emerged as a progressively significant domain of investigation and advancement in recent times. Particularly, the alteration of cell surfaces using meticulously crafted and thoroughly characterized synthesized molecules has proven to be an efficacious means of introducing innovative functionalities or manipulating cells. Within this realm, a diverse array of elegant and robust strategies have been recently devised, including the bioorthogonal strategy, which enables selective modification. This review offers a comprehensive survey of recent advancements in the modification of mammalian cell surfaces through the use of synthetic molecules. It explores a range of strategies, encompassing chemical covalent modifications, physical alterations, and bioorthogonal approaches. The review concludes by addressing the present challenges and potential future opportunities in this rapidly expanding field.

## Abstract 2

This review offers a survey of recent advancements in the modification of mammalian cell surfaces through the use of synthetic molecules and concludes by addressing the present challenges and potential opportunities in this rapidly expanding field.

## Graphical abstract

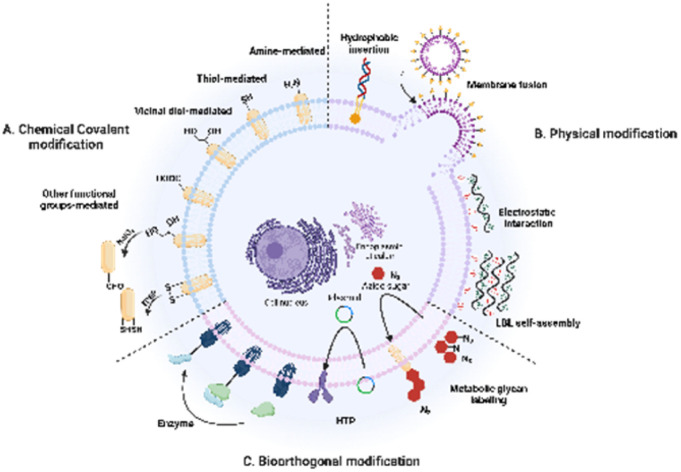

## 1. Introduction

Mammalian cells (hereinafter referred to as “cells”), as natural constituents of organisms, have been propelled into the spotlight in the biomedical field, primarily due to their unique characteristics, such as biosynthesis ability, communication and interaction ability, and migration ability, among others.^1–4^ Over the past few decades, the manipulation of cells has provided a powerful tool to enhance our understanding of the underlying mechanisms governing various biological behaviors in basic research and has also promoted the development in biomedical applications, such as medical diagnosis and cell-based therapy.^5^ For instance, mesenchymal stem cells (MSCs), red blood cells (RBCs), and macrophages exhibit promising capabilities as delivery vehicles to transport diagnostic molecules or therapeutic agents.^6–8^ Immune cells, including T cells and natural killer (NK) cells, have emerged as highly prominent candidates for tumor immunotherapy owing to their specific cytotoxicity against tumor cells while sparing normal cells.^9,10^

Despite these exciting achievements, the functions of natural cells themselves are limited. The cell surface, also known as the cell membrane, is a highly heterogeneous and dynamic milieu comprising lipids, proteins, carbohydrates, and their complexes, which governs numerous intracellular and extracellular processes.^11^ Simultaneously, the complex cell surface provides plenty of opportunities for further modification aimed at achieving particular functionalities, a process referred to as cell surface modification. This process serves as a powerful means to facilitate the biomedical application of natural cells.^12^ One notable example is the universal blood, which involves the modification of red blood cell surfaces to impede the recognition of antigenic sites by antibodies, thereby preventing immune responses caused by blood type incompatibility.^13–15^ Chemical manipulation of cell behavior and function through modification of cell surfaces using precisely synthesized and well-characterized synthetic molecules, such as polymers, is a captivating area of research.^16^ Various strategies have been devised for this purpose. While several reviews have provided summaries of certain strategies, there is a noticeable dearth of comprehensive discussions specifically centered on the strategy of cell surface modification using synthetic molecules.^16–18^

**Fig. 1:**
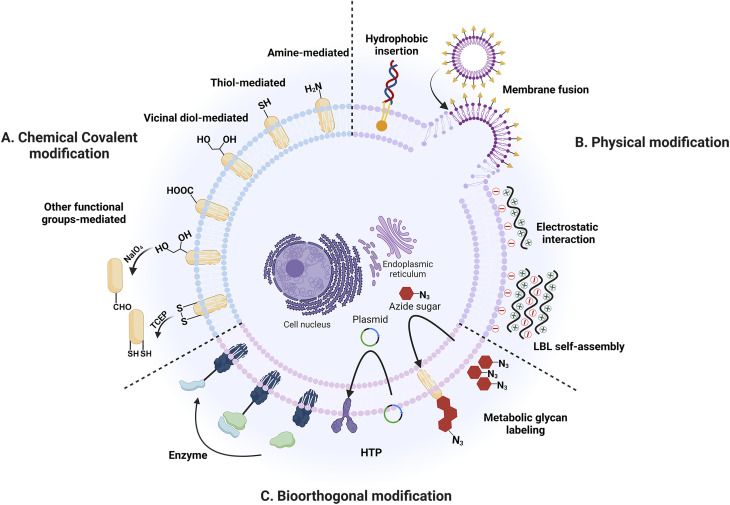
Mammalian cell surface modification strategies. (A) Chemical covalent modification strategies. (B) Physical modification strategies. (C) Bioorthogonal modification strategies.

Hence, this paper provides a comprehensive review of the latest advancements in strategies related to the modification of cell surfaces accompanied by a discussion on their biomedical applications, with a particular emphasis on developments from 2017 onwards. These strategies encompass a range of approaches, including chemical covalent approaches, physical techniques, and bioorthogonal methods for synthetic molecules (Fig. 1). The synthetic molecules discussed include specific functional groups, synthetic functional small molecules, synthetic polymers, synthetic nanoparticles, synthetic cell coatings, and synthetic DNA, among others. Lastly, the paper discusses the existing challenges and potential future prospects in this rapidly expanding field.

## 2. Chemical covalent modification

**Fig. 2:**
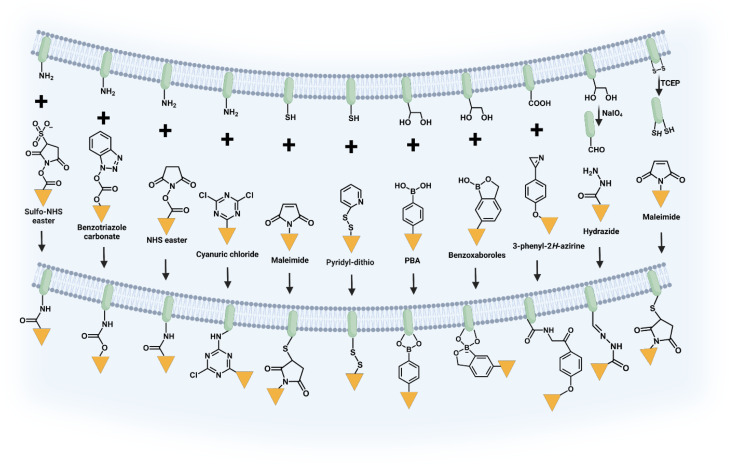
Illustration of chemical covalent modification through the reactions between functional groups on cells and synthetic molecules.

Chemical covalent modification is a strategic approach that entails the utilization of the chemically reactive functionalities found on the surface of the cell membrane. The cell membrane, a multifaceted chemical structure composed of lipids, proteins, carbohydrates and other components, offers a diverse array of functional groups that can be employed for chemical covalent binding.^19^ Previous researches have predominantly focused on employing amine, thiol, and vicinal diol groups present on amino acid residues within proteins or sugar residues as the most frequently utilized groups (Fig. 2).^20^ The stable attachment of synthetic molecules and the absence of cell pretreatment are the primary benefits of this approach, making it a simple yet effective method for modifying cell membranes. However, it is widely recognized that directly modifying cell membranes with reactive functional groups through covalent bonds can potentially impair the functionality of membrane proteins and subsequently impair cellular functions. Consequently, when employing this strategy, careful attention must be paid to both cell viability and effector functions.

### 2.1 Amine-mediated covalent modification strategy

Amine groups (–NH_2_) are mainly present at the lysine residues of proteins and the N-terminus of polypeptide chains. They are widely used for chemical modification of cell membrane surfaces due to their ease of chemical covalent modification and mild reaction conditions. The amine-mediated covalent binding strategy can be achieved through two primary pathways: acylation or alkylation. Generally, these reactions exhibit rapidity and selectivity, resulting in the formation of stable bonds (such as amide or secondary amine bonds) and high yields.

**Fig. 3:**
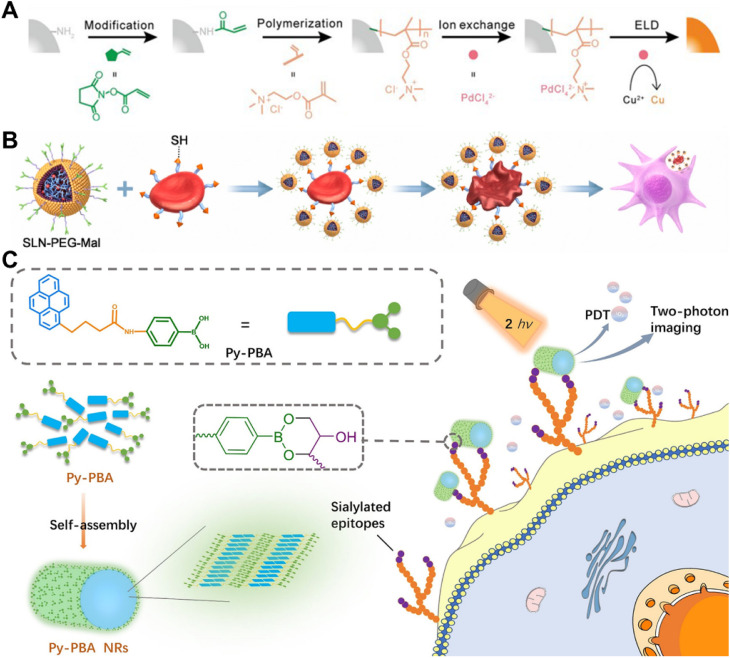
Methods based on chemical covalent modification. (A) Schematic illustration of realizing polymer-assisted cell metallization by the use of reactions between amino groups on cell membranes and NHS-AA.^22^ Copyright 2021, John Wiley & Sons, Inc. (B) Schematic illustration of modifying RBCs with SLN-PEG-Mal through reactions between maleimide and thiol groups, leading to their engulfment by macrophages.^36^ Copyright 2023, Elsevier. (C) Schematic illustration of combining Py-phenylboronic acid (PBA) NRs to the cell membrane by vicinal diol groups for two-photon imaging of cell surface sialic acids and PDT.^37^ Copyright 2021, American Chemical Society.

Among various kinds of reagents, *N*-hydroxysuccinimide (NHS) ester is the most frequently used to covalently bind to –NH_2_ on cell membranes. Recently, Cai *et al.* modified human umbilical vein endothelial cells (HUVECs) and human skin fibroblasts (HSFs) with succinimide ester-methoxy polyethylene glycol (NHS-mPEG), resulting in a significant enhancement of cell migration ability and motility through reduction of the focal adhesion area.^21^ In a separate study shown in Fig. 3A, Wang *et al.* employed acrylic acid NHS ester (NHS-AA) to immobilize vinyl onto cell membranes, followed by free radical polymerization to covalently attach polymers to the membrane.^22^ After subsequent ion exchange and electroless deposition (ELD), the polymer-functionalized cells could be converted into metallic biocomposites, which can be applied in the fields of biosensors, electronics, and energy. Sulfo-NHS ester is a more suitable reagent for covalent reactions with –NH_2_ groups on the cell membrane due to its enhanced water solubility and negative charge, which reduces the transmembrane permeability of the sulfo-NHS ester. For instance, Jasiewicz *et al.* employed sulfosuccinimidyl 4-(*N*-maleimidomethyl) cyclohexane-1-carboxylate (sulfo SMCC), a heterobifunctional crosslinker, to modify MSCs by covalently binding to the amines on the cell membrane and subsequently decorating them with heterodimerizing leucine zippers.^23^ In addition, sulfo-NHS-biotin was also commonly utilized to modify the cell membrane where it serves as a “bridge” and facilitates the decoration of the cell membrane with cargoes through streptavidin–biotin interaction.^24–29^ In addition to NHS ester derivatives, other types of reagents have been developed for covalently binding to –NH_2_ including cyanuric chloride and benzotriazole carbonate.^30,31^

### 2.2 Thiol-mediated covalent modification strategy

Thiol groups (–SH), mainly located on the cysteine residues of amino acids in proteins, are one of the most potent nucleophiles, stronger than amino groups. Thiol groups are frequently employed for the covalent modification of cell membranes. Maleimide derivatives, which form stable thioether bonds with thiol groups through an energetically favorable Michael addition reaction, are the most widely used reaction reagents because they exhibit high stability and chemoselectivity with thiol groups. The significant advantage of this strategy lies in the extensive availability of commercially accessible reagents and linkers.

Early research was conducted by Irvine's team which focused on surface modification of various cell types containing thiols. They investigated the application of liposomes and liposome-like nanoparticles containing maleimide terminal groups for T cells, hematopoietic stem cells, and other cell surface modifications.^32–36^ Recently, Wang *et al.* developed PEGylated solid lipid nanoparticles functionalized with maleimide end groups (SLN-PEG-Mal).^36^ As is shown in Fig. 3B, by exploiting the reaction between maleimide and sulfhydryl groups on the surface of RBCs, the researchers successfully enhanced the adsorption of modified nanoparticles onto RBCs, leading to significant alterations in the properties and morphology of RBCs. Moreover, these nanoparticle-loaded RBCs exhibited a remarkable ability to be engulfed by macrophages, thereby demonstrating promising potential for targeted drug delivery to macrophages. Wang *et al.* utilized 2-iminothiolane (Traut's agent), a thiolation reagent, to introduce extra free thiol groups by capping primary amines with thiol groups. This allows them to modify platelets with PD-L1 antibody, thereby reducing post-surgical tumor recurrence and metastasis.^35^

Research has been conducted to combine maleimide with other functional components in order to develop multifunctional nanoparticles.^38^ For instance, Luo *et al.* synthesized double-bound magnetic nanoparticles (DBMN) containing PEG-Mal, hyaluronic acid (HA), and Fe_3_O_4_.^38^ Following a simple incubation, DBMN was able to anchor onto the cell membrane through a Michael addition reaction between the Mal component and sulfhydryl groups on the T cell surface, resulting in magnetized T cells (DBMN-T). Under external magnetic field guidance, DBMN-T exhibited excellent targeting ability. Additionally, HA could bind to highly expressed CD44 on tumor cells, promoting recognition and killing of tumor cells.

In addition to the reaction between maleimides and thiols, cell surface modification could also be achieved through the exchange between disulfide bonds and thiols.^39–43^ Wayteck *et al.* incorporated thiol-reactive phospholipids into liposome bilayers with a pyridyldithiopropionate (PDP) head group, which was capable of forming reducible disulfide bonds with thiol groups exposed on the cell surface, thereby enabling the covalent coupling of liposomes.^39^

### 2.3 Vicinal diol-mediated covalent modification strategy

Vicinal diol groups are abundant on the cell membrane and primarily originate from sialic acid (SA), mannose, and galactose residues within glycoproteins and the extracellular matrix. Phenylboronic acid (PBA) derivatives form unique dynamic covalent bonds with vicinal diol groups of the cell membrane and are affected by pH, making them of substantial interest. However, only sialic acids can be efficiently coupled to PBA under physiological conditions, while other diol groups require alkaline reaction conditions with a pH value higher than the p*K*_a_ of PBA. Therefore, most studies have focused on the reaction between PBA derivatives and SA on the cell membrane surface. Tao and colleagues reported a novel fluorescent polymer containing PBA through the combination of multicomponent reactions (MCRs) with reversible addition–fragmentation chain transfer (RAFT) polymerization.^44,45^ Specifically, the Hantzsch reaction, a classical four-component reaction, was carried out simultaneously with RAFT polymerization to create innately fluorescent 1,4-dihydropyridine (1,4-DHP). The formed fluorescent polymer is suitable for cell membrane conjugation and imaging through the interaction between phenylboronic acid and sialic acid on the cell membrane. In our laboratory, we utilized a copolymer containing PBA groups to modify silicon nanowire arrays which exhibited a high capture capacity for cells overexpressing SA on the membrane. This modification also allowed for high efficiency of intracellular delivery of diverse biomacromolecules.^46^ Additionally, overexpression of SA has been demonstrated in various tumors, including lung, melanoma, colon, and breast cancers.^47^ Consequently, the SA-PBA reaction was commonly employed for tumor cell imaging, targeting, and capturing.^37,48–54^ Li *et al.* developed self-assembled nanorods of PBA-functionalized pyrene (Py-PBA NRs), which possess a highly efficient and specific imaging feature of SA on the cell membrane. Three cell lines with different expression levels of SA were utilized to demonstrate this imaging ability. Additionally, the nanorods exhibited efficient generation of ^1^O_2_ under two-photon irradiation, providing potential possibilities for tumor therapy (Fig. 3C).^37^ Furthermore, PBA derivatives with low p*K*_a_ values have been developed to enhance the applicability of this strategy to cell species characterized by low expression levels of sialic acids. A series of PBAs with different substituents were synthesized, and it was demonstrated that the introduction of electron-withdrawing groups, such as fluoro and nitro, effectively decreased the p*K*_a_ even to 4.2.^55^ However, it should be noted that the optimal binding pH may not always exceed the p*K*_a_ of PBAs, particularly in complex multicomponent systems.^56^

In addition to PBA derivatives, benzoxaborole (BA), a cyclic hemi-ester of boronic acid, can also be utilized for cell surface modification *via* the covalent reaction with vicinal diol.^57^ Morgese *et al.* have successfully modified supramolecular polymers containing BAs onto the surface of human RBCs *via* the covalent reaction between BAs and SA.^58^ The specific interactions between functional copolymers and the cell surface were further visualized in real time using total internal reflection fluorescence microscopy.

### 2.4 Other functional groups-mediated covalent modification strategy

Carboxyl groups are abundantly present on the cell membrane, mainly distributed at the residues of aspartic acid (Asp) and glutamic acid (Glu) within membrane proteins, as well as at the C-terminus of polypeptide chains. However, the modification of membranes using carboxyl groups necessitates pre-activation of these groups, typically employing an activator known as 3-(ethyliminomethylideneamino)-*N*,*N*-dimethylpropan-1-amine (EDC), which causes significant harm to mammalian cell viability. Consequently, the utilization of carboxyl groups for cell modification is often limited. Recently, Ma *et al.* developed a novel probe (3-phenyl-2*H*-azirine) that effectively labeled carboxyl groups on the surface of living cells. This presented new possibilities for chemical modification utilizing carboxyl groups present on the cell membrane.^59^

In addition to utilizing existing groups for direct modification of the cell membrane, strategies have been devised to convert commonly present but difficult-to-modify functional groups into easily modifiable ones through mild oxidation or reduction reactions on the cell surface under gentle conditions. For example, researchers have found that a mild oxidation reaction using NaIO_4_ can convert diols on cell surfaces into aldehydes, which can be treated as active sites for subsequent cell modification.^60–65^ Recently, Liu *et al.* proposed a novel cell surface engineering platform using classical thiazolidine chemistry to combine small molecules containing aminothiol moieties with cells pretreated with aldehyde groups on their surface by NaIO_4_.^64^

Efforts have also been devoted to converting disulfide bonds (S–S) on cell membranes to thiol groups and tris(2-carboxyethyl) phosphine (TCEP) is a widely used mild reducing agent in this strategy. Researchers have utilized the strategy to modify synthetic materials including chondroitin sulfate (CS), PEG, mesoporous silica nanoparticles, and silver nanoclusters on the cell membrane for a diverse range of applications.^66–70^

## 3. Physical modification

In addition to the chemical covalent binding strategy, physical approaches such as hydrophobic insertion, membrane fusion, electrostatic interaction, and layer-by-layer self-assembly offer versatile and easy ways to introduce synthetic molecules to the cell membrane while maintaining cellular physiology.

### 3.1 Hydrophobic insertion

**Fig. 4:**
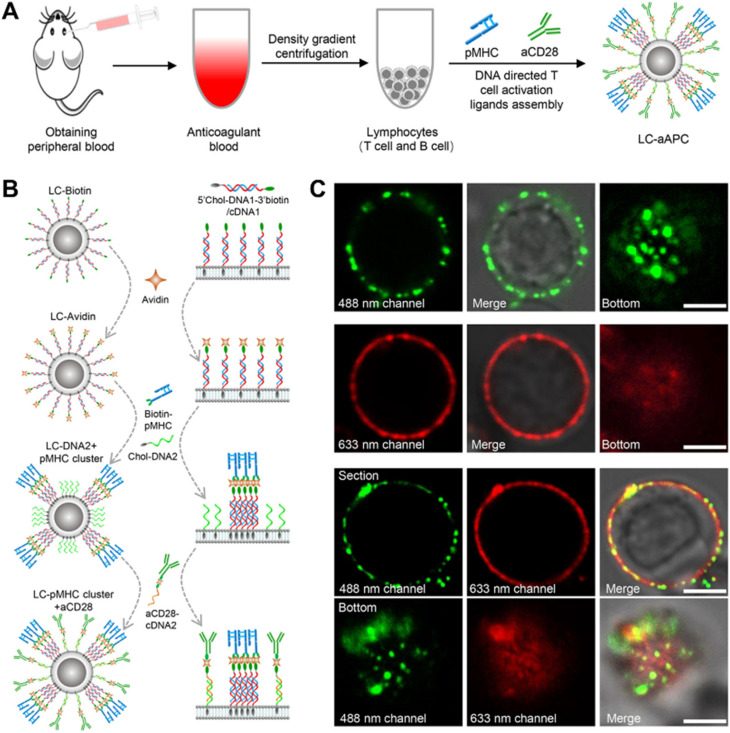
Construction of LC-aAPCs with lymphocytes from peripheral blood.^106^ (A) Schematic diagram of constructing lymphocyte-based aAPCs from peripheral blood for personalized tumor immunotherapy. (B) Schematic illustration of DNA-mediated bottom-up assembly of pMHC-I and aCD28 on lymphocytes, including hydrophobic insertion and specific DNA hybridization as well as biotin–avidin interaction. (C) Confocal microscope images showing distribution of pMHC-I and aCD28 on the surface of lymphocytes. Copyright 2022, John Wiley & Sons, Inc.

The cell membrane skeleton is a phospholipid bilayer structure allowing the spontaneous insertion of synthetic materials with hydrophobic anchors or “tails” driven by the hydrophobic effect.^71–73^ The commonly used hydrophobic anchors include lipids (phospholipids^21,26,73–80^ and cholesterols^81–92^), alkane chains^93–98^ and oleyl chains.^99–105^ They can be categorized into single and multiple anchors based on the number of hydrophobic anchors. Shi *et al.* constructed a polyvalent antibody mimic (PAM) for engineering NK cells with highly efficient targeting, adhesion and killing effects for tumor cells.^71^ The DNA initiator (DI) with a single anchor was displayed on the NK cell membrane by the hydrophobic insertion approach. Subsequently, a DNA scaffold was synthesized and hybridized with multiple aptamers *in situ* forming PAM-engineered NK cells. Sun *et al.* reported a DNA-assisted bottom-up self-assembly approach for achieving precise control over the lateral and vertical distributions of T cell activation ligands on RBCs and constructing RBCs-based artificial antigen presenting cells (aAPCs) which could effectively activate and expand T cells.^107^ DNA strands with a cholesterol end group were inserted into the membranes by hydrophobic interaction and then bound with T cell activation ligands through specific DNA hybridization as well as biotin–avidin interaction. The vertical distributions of T cell activation ligands can be easily manipulated by adjusting the length of DNA strands, while the lateral distributions were achieved through biotin–avidin interaction. The subsequent study shown in Fig. 4 employed the approach to construct lymphocyte-based aAPCs exhibiting homologous targeting functionality for personalized cancer immunotherapy.^106^ Zhao *et al.* developed a surface-anchored framework for sheltering the epitopes on Rhesus D (RhD)-positive RBCs.^102^ RBCs were modified with horseradish peroxidase containing a single oleyl chain *via* hydrophobic insertion, thereby catalyzing the reaction of H_2_O_2_ to construct a polysialic acid (PSA)-tyramine framework on the RBC membrane. The crosslinking framework successfully achieved transfusion of the modified RBCs to RhD-negative recipients without eliciting immunogenicity, by effectively balancing the modified fluidity of RBC membranes and shielding of RhD antigens.

In addition to the single anchor, hydrophobic insertion moieties with more anchors were developed. Niu *et al.* reported the first effort for cytocompatible controlled radical polymerization (CRP) techniques.^72^ Chain-transfer agents with a two-tailed hydrophobic anchor, 1,2-distearoyl-*sn*-glycero-3-phosphoethanolamine (DSEP), were successfully modified on the cell membranes with the hydrophobic insertion strategy, thereby realizing polymerization to be initiated directly in the live cell surface while maintaining high cell viability. The strategy effectively enhanced the efficiency of grafting polymers compared to the traditional grafting-to methods and offered novel possibilities for modulating cellular interactions.

**Fig. 5:**
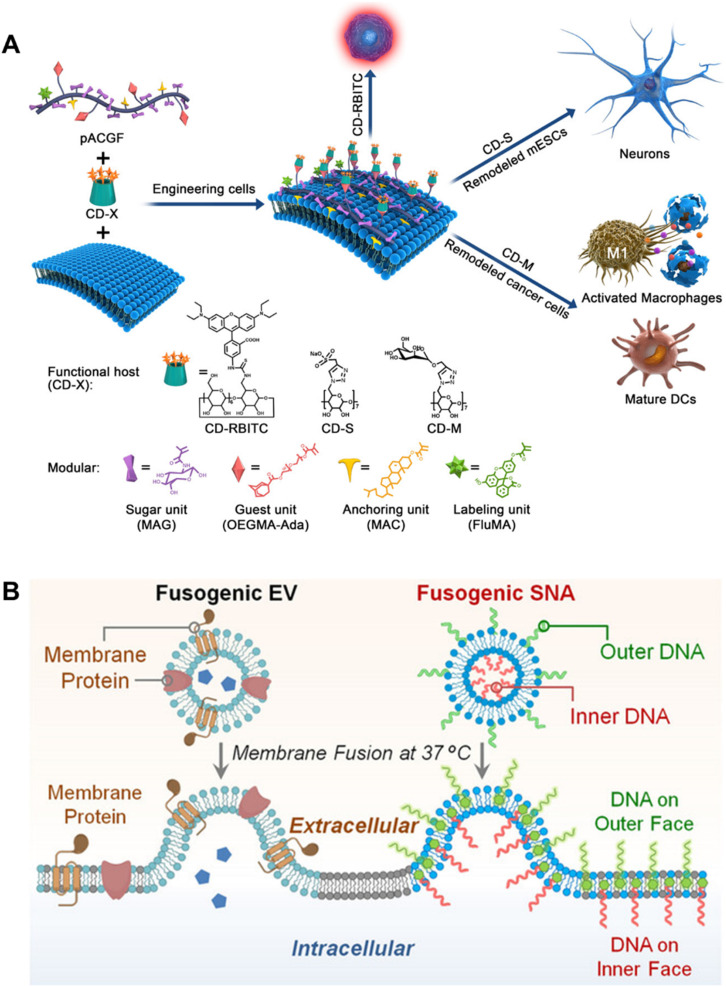
Representative examples based on hydrophobic insertion and membrane fusion strategies. (A) Schematic illustration of the construction of a platform for the manipulation of cell behaviors, by using hydrophobic insertion to bind modular polymers to the cell surface.^91^ Copyright 2019, American Chemical Society. (B) Schematic showing the biomimetic LiFT approach to engineer the plasma membrane by membrane fusion.^108^ Copyright 2022, John Wiley & Sons, Inc.

Additionally, the method was also utilized for T cell modification with liposomal nanoparticles, as described in Hao *et al.*'s investigation.^73^ The tetrazine (Tre) groups with two-tailed lipids (DSPE) were inserted into T cells and subsequently drug liposomes with bicyclo nonyne (BCN) were modified on the cell membranes of T cells *via* click reaction while preserving the intact functionality of T cells. A platform (Fig. 5A) for cell membrane engineering with modular polymers was developed by our group.^91^ The study employed cholesteryl-methacrylate as one of the monomers for constructing modular polymers with multiple anchors through one-pot RAFT copolymerization, along with deoxy-2-(methacrylamido)glucopyranose (MAG) as a hydrophilic monomer and adamantane carbonyl methacrylate (Ada) as a guest monomer. In addition to the introduction of functional molecules through host–guest units, the residence time of the modular polymers could also be regulated on the cell membrane by adjusting the content of cholesterol modules.

The hydrophobic insertion strategy is considered a simple, powerful, and less invasive approach for cell surface modification. However, functional synthetic materials introduced to the cell membrane surface through hydrophobic insertion are prone to loss during membrane flow and endocytosis, thereby limiting their long-term presence on the cell membrane. The hydrophobic insertion moieties with multiple anchors may offer a promising strategy for achieving relatively stable and long-time modification.

### 3.2 Membrane fusion

Unlike the strategy of hydrophobic insertion into the cell membrane through hydrophobic anchors, cell surface modification is achieved through liposomes loaded with synthetic materials or functional groups diffusing and mixing with the cell membrane in the membrane fusion strategy.

Sarkar *et al.* developed a versatile platform technology for the modification of cell membranes.^109^ Biotinylated lipid vesicles were utilized for the incubation with MSCs, leading to the attachment of biotin on the cell surface *via* vesicle fusion. The biotin moieties serve as binding sites for subsequent ligands. Yousaf and colleagues conducted a series of studies that utilized the membrane fusion strategy to introduce chemical functional groups onto the cell membrane.^110–118^ The prepared lipid, containing either ketone or oxyamine molecules, underwent spontaneous insertion and fusion into the cell membrane, resulting in the modification of cells with either ketone or oxyamine molecules for subsequent bio-orthogonal ligation reactions.^110^ Additionally, the bioorthogonal molecules, ketone or oxyamine, could also be modified onto different populations of cells using the same method to regulate the cell–cell interaction and generate 3D tissue-like structures.^111^ Following this, the researchers employed a membrane fusion approach to create and modify cell membrane surfaces with bioorthogonal chemical molecules possessing diverse characteristics, including photoresponsive and redox-responsive cleavage. The primary emphasis of their investigation was on the utilization of these modified cells in the field of three-dimensional tissue engineering. Membrane fusion strategies have recently been extensively used as a potent tool for modifying cell membranes in various investigations.

Zheng *et al.* designed core–shell membrane-fusing liposome (MFL) containing NK cell-activating glycans, Lewis X trisaccharide (LeX), and loaded it into a thermosensitive hydrogel which could be released responsively through the tumor microenvironment. Subsequently, the released MFL was fused with tumor cell membranes, realizing the modification of tumor membranes with Lex which could enhance the anti-tumor effects.^119^ Shi *et al.* designed T-cell-targeting fusogenic liposomes by conjugating ROS-scavenging groups, 2,2,6,6-tetramethylpiperidine (TEMP) and T-cell-targeting anti-CD3 F(ab′)_2_ fragments to the surface of liposomes, in which TEMP groups were designed for neutralizing ROS and protecting T cells from an oxidation-induced loss of activity. In the meantime, the procedure would result in paramagnetic transition of TEMP to TEMPO molecules, allowing for the measurement of the *in situ* activity of T cells, enabling a better understanding of engineering T cells for cancer treatment.^120^ Lin *et al.* reported a liposomal fusion-based transport (LiFT) strategy to anchor functional DNA strands on the inner face of the cell membrane, addressing the previous lack of suitable synthetic tools to engineer the intracellular interior (Fig. 5B).^108^ In subsequent studies, the group combined membrane-anchored catalysts with the previously reported LiFT strategy, through which they were able to prepare the corresponding fusion liposome catalyst through a simple strategy, achieving precise position control of the catalyst on the cell membrane.^121^ The drug molecules generated by this method may have higher drug delivery efficiency than traditional methods using drug delivery vehicles. Furthermore, by integrating targeting motifs into the outer surface of liposomes, cell-specific membrane engineering can be achieved for potential targeted drug delivery.

### 3.3 Electrostatic interaction

Cell surface modification through electrostatic interactions is an appealing strategy that capitalizes on the negative charge conferred mainly by sialic acid residues in the carbohydrate layer, along with the phosphatidylserine on the plasma membrane. Cationic polymers such as polyethyleneimine (PEI), poly-l-lysine (PLL) and chitosan (CS) are often utilized in the strategy.^122–126^ For instance, Choi and colleagues achieved the development of silica coating on mammalian cells by modifying PEI on the cell membrane through electrostatic interactions, serving as a catalytic template for silicification.^126^ In a subsequent study, TiO_2_ shells were developed for the cytoprotective encapsulation of Jurkat T cells.^122^ This method could effectively protect the T cells in the shell while simultaneously preserving their functionality, including cell division, juxtacrine interactions and cytokine secretion. Upon administration into the organism, the lymphocytes' therapeutic capabilities are effectively reinstated through the rupture of the protective shell. The TiO_2_-inducing peptide, (RKK)_4_D_8_ (R: arginine, K: lysine, D: aspartic acid), was deposited on the surface of Jurkat cells *via* electrostatic interactions to facilitate the formation of bioinspired TiO_2_ using titanium bis(ammonium lactato)dihydroxide (TiBALDH) as a precursor. However, interactions with most cationic polymers readily lead to the destruction of the cell membrane, resulting in pronounced cytotoxicity and cellular damage. To address this issue, cationic polymers can be modified with biocompatible molecules, such as grafting PEG or alginate, to mitigate the detrimental effects on cell viability.^127^

Despite the overall negative charge of the cell membrane surface, a few cationic sites on the plasmalemma still exist which can be modified with negatively charged materials.^130,131^ For instance, Thomsen *et al.* modified T cells with negatively charged degradable poly(lactic acid) (PLA) nanoparticles with electrostatic adsorption.^125^ Furthermore, the modification of negatively charged materials can be achieved through a synergistic combination of electrostatic interactions, hydrogen bonding and hydrophobic interactions.^132–134^ It is important to note that the presence of a negatively charged cell membrane hinders the uptake of negatively charged nanoparticles by cells.

### 3.4 Layer-by-layer (LBL) self-assembly

**Fig. 6:**
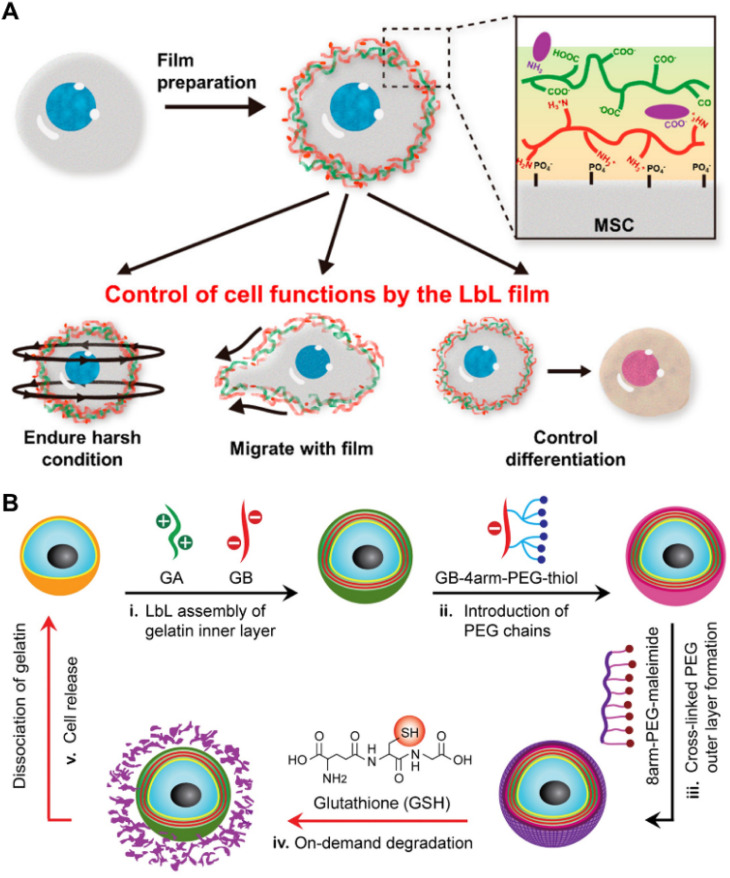
Methods based on layer-by-layer self-assembly. (A) Schematic illustration of MSCs nanofilms prepared using positively charged PLL, negatively charged HA and RGD, and the functions exhibited by the modified cells.^128^ Copyright 2017, American Chemical Society. (B) Schematic illustration of mammalian cell nanoencapsulation conducted by LBL self-assembly between GA and GB, and subsequent thiol-maleimide reaction. GSH could be added for on-demand release.^129^ Copyright 2017, Elsevier.

LBL self-assembly strategies have been developed on the basis of electrostatic interaction and widely employed for the construction of cell coatings, in which oppositely charged materials are sequentially deposited onto the cell membrane through electrostatic interaction along with hydrogen bonding, van der Waals forces, *etc.*^21,127,128,135–147^ As shown in Fig. 6A, Hong and colleagues developed LBL self-assembled nanofilms for cell surface modification of viable MSCs. Positively charged PLL was layer-by-layer assembled with negatively charged hyaluronic acid (HA) and arginine-glycine-aspartic acid (RGD) to fabricate nanofilms, which not only provided biochemical signals but also offered mechanical support for MSCs without interfering with the stemness of MSCs.^128^ Subsequent studies have demonstrated the successful construction of nanofilms on the surface of human induced pluripotent stem cells (iPSCs) and immune cells such as AML-12 cells and peripheral blood mononuclear cells (PBMCs) *via* the LBL self-assembly strategy.^140,148^ Gels can be formed *via* LBL to coat or encapsulate cells. Chen and colleagues proposed a gentle approach (Fig. 6B) to achieve the nanoencapsulation of individual mammalian cells.^129^ The gelatin coatings, which mimic the extracellular matrix (ECM), are formed through LBL self-assembly between positively charged gelatin type A (GA) and negatively charged gelatin type B (GB) on the cell membrane surface. Additionally, the outer layer of PEG was further constructed using thiol-maleimide click chemistry which could be degraded on-demand by the addition of the reducing agent glutathione (GSH). Subsequent studies involved the development of an enzyme-responsive nano-coating for encapsulating individual living cells, which was prepared through layer-by-layer self-assembly of oppositely charged gelatin-poly(ethylene glycol)maleimide and the incorporation of cysteine-terminated peptide sequences (CGGPLGLAGGC) *via* click reaction.^135^ Moreover, the peptide chain could undergo enzymolysis upon exposure to high concentrations of matrix metalloproteinase-7 (MMP-7), which is frequently overexpressed in tumors, leading to the release of encapsulated cells.

## 4. Bioorthogonal modification

Despite the abundance of functional groups on the surface of the cell membrane that are amenable to chemical covalent modification, the utilization of non-specific covalent modification strategies may have detrimental effects on the viability and functionality of normal cells. Additionally, physical strategies are limited by the short residence time of synthetic molecules. In contrast, bioorthogonal chemistry offers a highly efficient and selective approach that takes place within a mild physiological environment, without disrupting intrinsic biochemical processes. This strategy represents a substantial advancement in terms of both cell viability and the stability of modifications. Here, we provide an overview of recent developments in the integration of metabolism with copper-free click chemistry, Halo-Tag proteins, and enzyme-mediated approaches.

### 4.1 Metabolic glycan labeling strategy

**Fig. 7:**
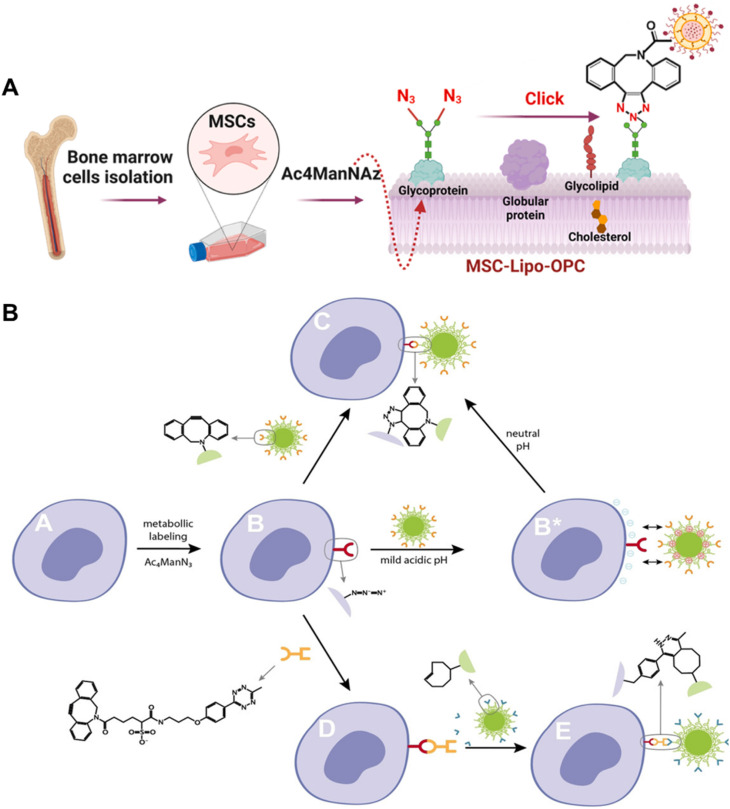
Methods based on the metabolic glycan labeling strategy. (A) Schematic illustration of binding Lipo-OPC to MSCs which were pre-incubated with Ac_4_ManNAz and presented azido groups on the cell membrane.^160^ Copyright 2023, Elsevier. (B) Overview of the 2-step click strategy for achieving highly specific cell surface conjugation of nanoparticles.^162^ Copyright 2020, John Wiley & Sons, Inc.

Since the pioneering work of Bertozzi and colleagues, who introduced exogenous glycans into the cell membrane glycocalyx, there has been a gradual development of strategies for metabolic glycan labeling to modify the cell membrane.^149,150^ In this strategy, unnatural sugars containing functional groups such as azide, alkyne, thiol and alkene are internalized by cells and the chemically reactive functional groups are “installed” on the glycan residues on the cell membrane by metabolic pathways.^150–156^ In recent years, glycans containing azide groups represented by *N*-azidoacetylmannosamine-tetraacetate (Ac_4_ManNAz) have gained the most widespread adoption with the development of copper-free “click” azide–alkyne reactions due to their high selectivity, synthetic simplicity and commercial availability. Tomas and colleagues have conducted a series of investigations on the cell surface modification with polymers *via* the metabolic glycan labeling strategy.^157–159^ In a recent study, Tomas *et al.* who proposed the “engineering cells to capture polymers” strategy incubated tumor cells with Ac_4_ManNAz for 96 hours to obtain azido-modified cancer cells which could capture chemotherapeutic polymers covalently and this strategy significantly augmented the concentration specifically targeted towards the tumor cell membrane whilst optimizing therapeutic efficacy by reducing systemic toxicity and enhancing selectivity.^158^ In addition to polymers, the metabolic glycan labeling method effectively facilitates the modification of nanoparticles on the live cell membrane which is an attractive strategy for drug delivery.^160–162^ Zhou *et al.* successfully modified an oligomeric proanthocyanidin loaded liposome on the membrane of MSCs (MSC-Lipo-OPC) *via* metabolic labeling combined with the click chemistry strategy (Fig. 7A).^160^ The MSC-Lipo-OPC could control the progression of inflammation due to the excellent abilities to scavenge free radicals and effectively prevent the formation of radiation-induced pulmonary fibrosis. Chen *et al.* developed polyvalent spherical aptamer (PSA) engineered macrophages which could effectively recognize tumor cells and inhibit tumor growth.^161^ PSA which has superior affinity and specificity to tumor cells was constructed through covalent reaction of gold nanoparticles (AuNPs) with AS1411 aptamer and DBCO groups, and was subsequently modified on macrophage membranes *via* metabolic labeling. Moreover, Lamoot *et al.* developed a 2-step click strategy for achieving highly specific cell surface conjugation of nanoparticles. In the study, cells were incubated with *N*-azidoacetylmannosamine-tetraacetylated (Ac_4_ManN_3_) to present azido groups on cell membrane (Fig. 7B).^162^ Subsequently, sulfo-6-methyl-tetrazine-dibenzyl cyclooctyne (Tz-DBCO) was exploited as a “bridge” between azide-modified cells and *trans*-cyclooctene (TCO) functionalized nanoparticles to realize nanoparticle-engineered cells exhibiting extremely low non-specific background binding.

In addition to *in vitro* applications for modifying cell membranes, researchers have also conducted studies to achieve this process *in vivo*.^163,164^ Wang *et al.* conducted an interesting study by labeling and modulating DCs and regulating DC–T cell interactions *in vivo*.^163^ They synthesized Ac_4_ManAz nanoparticles overcame the limitations of Ac_4_ManAz utilized *in vivo* such as poor encapsulation and water solubility. The Ac_4_ManAz nanoparticles and granulocyte-macrophage colony-stimulating factor (GM-CSF) were loaded into an injectable alginate gel for the purpose of realizing *in situ* recruitment and azide labeling of dendritic cells. Dibenzocyclooctyne (DBCO)-labelled immunomodulatory agents, such as tumor antigens, adjuvants, and cytokines could be modified on the DC membrane *via* click chemistry *in vivo* thereby effectively enhancing the subsequent T cell activation and tumor killing process. Additionally, Tu *et al.* employed Ac_4_ManAz nanoparticles for *in situ* labeling of tumor cell membranes with azido groups, followed by the binding of chlorin e6 (Ce6), a commonly used photosensitizer, *via* click chemistry.^164^ This approach effectively enhanced the therapeutic efficiency of photodynamic therapy. Recently, Chen and colleagues reported a cell-type-specific labeling approach *in vivo*.^165^ In this study, the cardiomyocyte was specifically labeled without any interference from other cardiac cell types, which provided a powerful tool for cell-type selective modification. Gong *et al.* accomplished *in situ* PEGylation of CAR-T cells through the utilization of the metabolic glycan labeling strategy.^166^ When the molecular weight of PEG reached 600000, it effectively hindered the intercellular interactions among CAR-T cells, tumor cells, and monocytes, thereby attenuating the secretion of cytotoxic cytokines and ameliorating the symptoms associated with cytokine release syndrome (CRS).

Compared to the direct covalent binding with functional groups on the cell membrane, the metabolic glycan labeling strategy effectively enhances the density of reactive sites on cells, but it is a time-consuming process that can take several days. The significant advantage of metabolic glycan labeling combined with bioorthogonal reactions is that it transits cell surface modification from nonspecific to specific, enabling cell surface modification *in situ* and *in vivo*. This is still an emerging field, with immense potential for further development and expansion.

### 4.2 Halo-Tag protein

Halo-Tag protein (HTP) is an engineered protein derived from the bacterial haloalkane dehalogenase, which selectively reacts with alkanes containing a terminal chloride group (chloroalkanes) forming a covalent bond.^167,168^ Similar protein recognition tags, such as SNAP tags^169^ and ACP tags,^170^ could also be utilized in cell surface modification, but they will not be extensively discussed in this section. A two-step approach was utilized in the HTP strategy: the expression of HTP on the cellular membrane is achieved *via* genetic engineering methods and further combined with cargoes containing the chloroalkane. HTP is commonly used in protein isolation and purification, molecular imaging, molecular interactions *etc.* in most reported studies and was first utilized for cell surface modification by Pulsipher *et al.*^171^ They proposed a long-lived cell membrane engineering strategy utilizing HTP as an anchor for modifying embryonic stem cells (ESCs) with heparan sulfate (HS), which was covalently modified on the ESC membrane and stayed for more than one week.

**Fig. 8:**
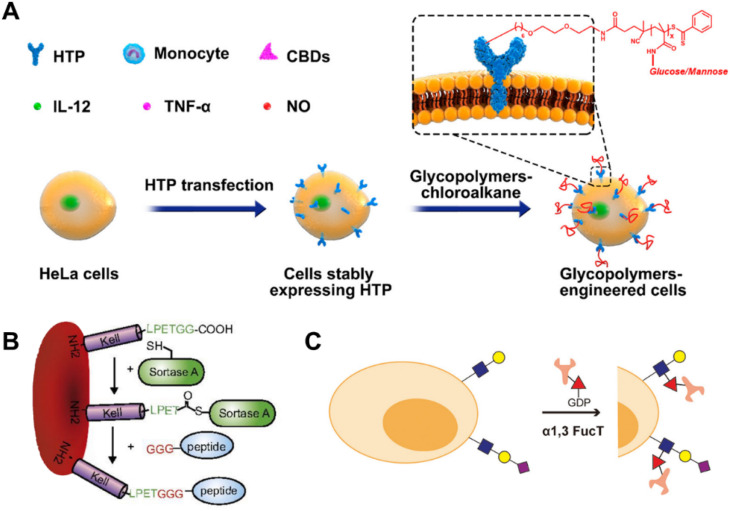
Methods based on other bioorthogonal strategies. (A) Schematic illustration of displaying synthetic glycopolymers on HeLa cell membranes using HTP anchors.^172^ Copyright 2019, American Chemical Society. (B) Schematic illustration of Kell C-terminal sortase labeling with GGG-carrying antigen peptides.^173^ Copyright 2017, National Academy of Sciences. (C) Schematic illustration of transferring biomacromolecules to glycocalyx on the surface of living cells with fucosyltransferase.^174^ Copyright 2018, American Chemical Society.

Subsequently, our group developed a series of studies *via* the HTP strategy.^172,175,176^ Tumor cells were modified with specific glycopolymers *via* the HTP fusion technique combined with RAFT polymerization (Fig. 8A). The glycopolymers that were modified on tumor cells could bind to lectins on dendritic cells or macrophages which effectively enhanced the tumor immune response.^172^ An interesting discovery was that the migration of the tumor cells modified with glycopolymers could be affected. Specifically, compared with the unmodified tumor cells, the migration direction was altered and diffusion slowed down which offered novel insights pertaining to the management of cancer metastasis.^175^ A following study was carried out and we constructed glycopolymers modified DCs *via* the HTP strategy. Enhanced interactions were discovered between glycopolymer modified DCs and T cells which effectively promoted the T cell activation and proliferation, providing a novel approach to designing more efficient DC vaccines.^176^

The HTP strategy for cell surface modification is still in its infancy. It is noteworthy due to the strong stability of binding between HTP expressed on the cell membrane and its corresponding ligand, thereby enabling sustained modifications that persist for over a week, offering a suitable method for long-time and stable cell surface modification. However, the implementation of HTP expression necessitates the manipulation of gene transfection, a process that is intricate and time-consuming, thereby inapplicable to certain challenging-to-transfect cell types such as primary cells.

### 4.3 Enzyme-mediated strategy

Enzyme-mediated modification of cell membranes represents a novel approach for *in situ* modification of candidate materials, reacting with pre-existing structures on the cell surface under the specific catalysis of enzymes. Specifically, certain enzymes such as oxidoreductases (galactose oxidases^177^), glycosyltransferases (sialyltransferases,^178,179^ galactosyltransferases, *N*-acetyl-glucosaminyl transferases and fucosyltransferases^174,180^), transpeptidases (butelases and sortases^173,181,182^), transglutaminases (TGases^183^) *etc.* have been utilized for the modification of cell membranes, representing an appealing approach due to their remarkable specificity and high yield. For example, galactose oxidases can specifically convert the endogenous terminal galactoses or *N*-acetylgalactosamine residues on the cell surface into aldehyde groups, facilitating subsequent reactions between aldehyde groups and aminooxy-functional molecules.^177^ Glycosyltransferases are primarily utilized for the modification of pre-existing sugars on cell membranes, thereby facilitating the introduction of non-natural sugars. Moreover, it's worth noting that in comparison to metabolic engineering approaches, glycosyltransferases, particularly sialyltransferases and fucosyltransferases, offer a novel method for introducing greater kinds and intricacy of sugars on the cell membrane surface. In the presence of transpeptidases, molecules bearing recognition motifs can be directly conjugated to either the N or C termini of membrane proteins. For example, as shown in Fig. 8B, Pishesha *et al.* reported a strategy for inducing antigen-specific tolerance by utilizing the transpeptidase sortase to covalently conjugate disease-associated autoantigens onto red blood cells (RBCs), thereby attenuating the contribution of major subsets of immune effector cells to immunity in an antigen-specific manner.^173^ Li *et al.* focused on fucosyltransferase and transferred bio-macromolecules to the glycocalyx on the surface of living cells, which represented faster speed, better biocompatibility, and less interference to cells (Fig. 8C).^174^ Through this method, they constructed two antibody–cell conjugates, which exhibited significant improvements in the process of targeting and killing anti-cancer immune responses.

## 5. Conclusion and outlook

Modifying cell surfaces with tailor-made and well-characterized synthesized molecules can effectively introduce novel functionalities or manipulate cells. This offers a powerful tool to overcome challenges encountered in cell-based biomedical applications. In this review, we present a comprehensive overview of the latest advances in cell surface modification using synthetic molecules. We summarize the typical strategies, including chemical covalent modifications, physical alterations, and bioorthogonal approaches (Table 1), along with the advantages, disadvantages, and applicable conditions of each strategy. The chemical covalent strategy offers a straightforward and versatile approach for achieving stable and long-lasting surface modification.^184^ However, the strategy has the potential to adversely impact cell activity and functionality. The physical modification strategy provides a non-invasive and cytocompatible approach. However, modifications achieved through physical interactions, such as electricity and hydrophobicity, are relatively short-term and unstable. It is important to note that the two methods mentioned above are non-specific, lacking precision in cell surface modification and potentially increasing the risk of adverse effects during practical applications. Therefore, bioorthogonal chemistry provides a valuable strategy for the selective and highly biocompatible incorporation of synthetic molecules onto cell surfaces, even enabling cell surface modification *in vivo* – a remarkable development. However, the approaches used to introduce bioorthogonal groups, whether *via* genetic engineering or metabolic engineering, are time-consuming.

**Table 1:** A summary of the advancements in strategies for the cell surface modification with synthetic molecules in this review

	Strategy	Cell type^a^	Synthetic molecules modified on the cell surface
Chemical covalent modification strategies	Amine groups (–NH_2_)	HUVECs	PEG,^21^ biotin–streptavidin^22^
		C2C12 cells	Poly(methacryloyloxy)ethyltrimethylammonium chloride (PMETAC)^22^
		MSCs	Heterodimerizing leucine,^23^ chitosan nanoparticles,^24^ sialyl Lewis X^29^
		RBCs	Biotin–streptavidin,^26^ PEG^30,31^
		T cells	Nanoparticles^32^
		Jurkat and NK cells	Biotin–streptavidin^27^
	Thiol groups (–SH)	RBCs	Nanoparticles^36^
		DCs	Bovine serum albumin (BSA) protein shells^41^
		B16 cells	BSA protein shells^41,43^
		T cells	Nanoparticles^33,34,38,39^
	Vicinal diol groups	L929 cells	Fluorescent polymer^44^
		MCF-7, HepG2 and HeLa cells	Pyrene derivative^37^
		B16 cells	Gadolinium DO3A amide^48^
		HDFs	DEGMA or NVP polymers^49^
		HepG2 cells	BSA nanoparticles^50^
		B16, AB22 and ZL34 cells	Contrast agents (Gd- and ^68^Ga-DOTA-EN)^52^
		PC-3, DU145 and Jurkat cells	Sialic acid-imprinted fluorescent core–shell SiO_2_ particles^53^
		MCF-7, HeLa and PC-3 cells	Polymer nanoparticles^54^
		RBCs	Supramolecular polymers^58^
	Carboxyl groups	MCF-7 cells	Reactive probe^59^
	Convert diols into aldehydes	HDFs	Biotin–streptavidin^60^
		HT29 and MDA-MB-231 cells	Maltol hydrazide^61^
		HepG2 cells	Dendrimer hydrazides^62^
			PEI conjugated with multiple hydrazide groups^63^
		MCF-7 cells	Peptide and protein^64^
		HeLa cells	*p*-Benzoquinone/ethylenediamine polymer^65^
	Convert disulfide bonds (S–S) to thiol groups	Human iPSC-derived-MSCs	ECM coating^66^
		HepG2 cells	DNA bridge complex-templated silver nanoclusters (DNA bridge-AgNCs)^67^
		HeLa cells	Fluorescent dye, polymer, and nanoparticles^69^
		NEs	Liposomal stimulator of interferon genes (STING) agonists^70^
Physical modification strategies	Hydrophobic insertion	HUVECs and HSFs	PEG^21^
		T cells	Tetrazine^73^
		DCs and PC-3 cells	Cucurbit[7]uril-based supramolecular polymer^74^
		CCRF-CEM cells, splenocytes, melanoma, human MSCs and beta cells	Synthetic peptide^76^
		RAW264.7 cells and L-O2 cells	β-Cyclodextrin (β-CD) and adamantane (ADA)^77^
	Hydrophobic insertion	Human T cells and B cells, erythrocytes, hepatocytes, L929 and HEK293T cells	Cell-penetrating peptide^79^
	Hydrophobic insertion	MCF7, A549, LUDLU-1 cells	Aptamer^80^
		HeLa cells	DNAzyme,^82^ DNA,^89^ multicomponent polymer,^91^ aptamer,^80^ core–shell upconversion nanoparticles^92^
		MCF-7 cells	Fluorescein isothiocyanate (FITC),^83^ core–shell upconversion nanoparticles^92^
		SubT1 cells	Biotin–PEG^84^
		MDA-MB-231 cells	Nitrilotriacetic acid (NTA)^85^
		NK cells,^81^ Jurkat, NK, and Ramos cells,^86^ CCRF-CEM cells,^87^ U937 cells,^88^ RBCs,^107^ lymphocytes^106^	DNA
		MSCs	Hyperbranched polyglycerol (HPG) covalently modified with vasculature binding peptides (VBPs)^93^
		Th9 cells, 4T1 cells	Peptides^94,95^
		Jurkat cells	ssDNA^97^
		3T3 cells	Poly(oxanorbornene) block copolymers,^98^ PEG^104^
		HepG2 cells	Peptides,^99^ PEG^100^
		RIN cells	PEG^101^
		Fibroblast cells	Ketone and oxyamine,^110,116,118^ dialdehyde,^112^ hydroquinone^117^
		NK cells	Lewis X trisaccharide^119^
		T cells	2,2,6,6-Tetramethylpiperidine groups^120^
		HeLa cells	DNA^108^
	Electrostatic interaction	HUVEC	Poly-l-lysine (PLL)^21^
		Jurkat cells	TiO_2_ coating^122^
		hASCs	Chitosan derivative^124^
		HeLa, 3T3, and Jurkat cells	Poly(ethyleneimine) (PEI)^126^
		T cells	PLA nanoparticles^125^
	Layer-by-layer (LBL) self-assembly	HeLa cells	Cationic gelatin and anionic gelatin^129,135^
		MSCs	PLL, hyaluronic acid (HA), and arginine-glycine-aspartic acid (RGD),^128^ gelatin,^135^ silk fibroin polyelectrolyte^139^
		RBCs	Chitosan-*graft*-phosphorylcholine, HA and PLL-PEG^136^
		DPCs	Gelatin and alginate^137^
		MIN6 cells	Gelatin and fibronectin (FN)^138^
		L929 cells	Silk fibroin polyelectrolyte^139^
		AML-12 cells and PBMCs	PLL and HA^140^
		T cells	Chitosan and alginate^147^
		iPSCs	FN, heparin (Hep), and chondroitin sulfate (CS)^148^
Bioorthogonal strategies	Metabolic glycan labeling strategy	Jurkat cells	Probes,^151^ lipid nanoparticles,^162^ polymer–antibody conjugates^156^
		HEK 293T cells	Probes^154^
		MCF-7 cells	β-CD and aptamer^155^
	Metabolic glycan labeling strategy	A549 cells	Poly(hydroxyethyl acrylamide),^157^ polycation,^158^ poly(hydroxyethyl acrylamide) (pHEA)^159^
		MCF-7 cells	Polycation^158^
		MSCs	Antioxidant liposome^160^
		Macrophages	Polyvalent spherical aptamer^161^
		DCs	Antigens, adjuvants and cytokines^163^
		4T1 cells	Chlorin 6 (ref. 164)
		HeLa cells	Probes^183^
	Halo-Tag protein	B16 cells,^175^ DCs,^176^ Hela cells^172^	Glycopolymers
	Enzyme-mediated strategy	B cells	Biotin^177^
		NK cells	Bio-macromolecules,^174^ Sialyl Lewis X and CD22-specific ligands^185^
		T cells	Bio-macromolecules^174^
		Mouse lung endothelial cells	Synthetic ligands and biotin^178^
		CHO cells	Probes,^181^ bio-macromolecules^174^
		RBCs	Probes,^182^ peptide^173^
		HEK 293T cells	Probes^181^
		HeLa cells	Probes^183^

a Abbreviation for the full cell name or cell species: HUVECs: human umbilical vein endothelial cells; C2C12 cells: murine myoblasts; MSCs: mesenchymal stem cells; RBCs: red blood cells; Jurkat cells: acute T-cell leukemia cells; NK cells: natural killer cells; DCs: dendritic cells; B16 cells: murine melanoma cells; L929 cells: mouse fibroblast cells; MCF-7 cells: human breast adenocarcinoma cells; HepG2 cells: human liver cancer cells; HeLa cells: human cervical cancer cells; HDFs: human dermal fibroblasts; AB22 cells: mouse mesothelioma cells; ZL34 cells: human mesothelioma cells; PC-3 and DU145 cells: human prostate cancer cells; iPSCs: induced pluripotent stem cells; HT-29: human colon cancer cells; MDA-MB-231 cells: human breast cancer cells; NEs: neutrophils; HSFs: human skin fibroblasts; CCRF-CEM cells: human acute lymphoblastic leukemia cells; RAW 264.7 cells: mouse leukemia cells of monocyte macrophages; L-O2 cells: human normal liver cells; HEK293T cells: human embryonic kidney cells; A549 cell: human lung cancer cells; LUDLU-1 cells: human Caucasian lung squamous carcinoma cells; SubT1 cells: human CD4 expressing T-lymphoblastoid cells; U937: histiocytic lymphoma cells; Th9 cells: helper T cell 9; 4T1 cells: mouse breast cancer cells; 3T3 cells: mouse embryonic fibroblasts; RIN cells: mouse insulinoma cells; hASCs: human adipose-derived mesenchymal stem cells; DPCs: dermal papilla cells; MIN6 cells: pancreatic β-cells; AML-12 cells: human lung cancer cells; PBMCs: peripheral blood mononuclear cells; CHO cells: Chinese hamster ovary cells.

Despite notable advancements in the utilization of synthetic compounds for cell surface modification, there remain unresolved challenges and prospects for further investigation. One such challenge pertains to the inherent detrimental impact of exogenous synthetic compounds bound to the cell surface on cellular functionality, albeit with varying degrees of severity. Hence, it is of utmost importance to meticulously choose a suitable strategy for modifying cells, taking into consideration the particular cell type and application scenarios. Subsequently, it becomes imperative to assess and describe the condition of the modified cells, encompassing cell viability, phenotype, and associated functionalities. It is noteworthy that the cell surface constitutes a dynamic membrane structure, wherein synthetic molecules may undergo endocytosis or excretion by the cell. Consequently, it is crucial to monitor the destiny of synthesized molecules during and post cell surface modification. In practical scenarios, the presence of synthetic molecules on cellular surfaces carries the potential for immune activation and subsequent clearance by the immune system, thereby considerably restricting their capacity to modify cell surfaces *in vivo*. Consequently, it becomes crucial to implement suitable adjustments to synthetic molecules to ensure their compatibility with *in vivo* applications. Additionally, a significant hurdle lies in selecting and designing molecules that possess both biocompatibility and augmented functionality for specific applications. Determining the optimal chemical group, structure, and sequence becomes essential in this regard. Therefore, the availability of databases serving as a toolbox for researchers to facilitate informed molecule selection is highly desirable.

There are several possible avenues for future research. One potential area of exploration is the development of more precise and targeted methods for modifying cell surfaces. There is a need to develop more proficient and potent methodologies for the selective and highly biocompatible integration of synthetic molecules onto cell surfaces. Furthermore, additional research is necessary to gain a better understanding of the influence of synthetic molecules on cellular functionality and to optimize modification strategies tailored to specific cell types and applications. An additional area of research that holds promise for the future is the advancement of synthetic molecules that possess improved biocompatibility and biofunctionality, enabling their application in the modification of cell surfaces. Notable examples of these molecules encompass functional nucleic acids, targeting aptamers, and polymers characterized by well-defined structures and chain sequences. The utilization of artificial intelligence (AI) can be facilitated by the establishment of databases containing comprehensive information regarding ligand–receptor interactions specific to cells, as well as the attributes associated with each modification technique. This integration of AI can aid in the design of optimal, customized molecules and the selection of appropriate methods for modification.

## Author contributions

Conceptualization: H. Chen, G. Chen and H. Yang; writing the original draft: H. Yang, L. Yao and Y. Wang; validation: H. Yang, L. Yao and Y. Wang; review and editing: H. Chen and G. Chen.

## Conflicts of interest

There are no conflicts to declare.

## Supplementary Material
